# Differentiation-Driven Nucleolar Association of the Mouse Imprinted *Kcnq1* Locus

**DOI:** 10.1534/g3.112.004226

**Published:** 2012-12-01

**Authors:** Andrew M. Fedoriw, J. Mauro Calabrese, Weipeng Mu, Della Yee, Terry Magnuson

**Affiliations:** Department of Genetics, Carolina Center for Genome Sciences, Lineberger Comprehensive Cancer Center, University of North Carolina at Chapel Hill, Chapel Hill, North Carolina 27599

**Keywords:** polycomb, noncoding RNA, stem cells, trophectoderm, differentiation

## Abstract

The organization of the genome within the mammalian nucleus is nonrandom, with physiologic processes often concentrated in specific three-dimensional domains. This organization may be functionally related to gene regulation and, as such, may play a role in normal development and human disease processes. However, the mechanisms that participate in nuclear organization are poorly understood. Here, we present data characterizing localization of the imprinted *Kcnq1* alleles. We show that nucleolar association of the paternal allele (1) is stimulated during the differentiation of trophoblast stem cells, (ii) is dependent upon the *Kcnq1ot1* noncoding RNA, (3) does not require polycomb repressive complex 2, and (4) is not sufficient to preclude transcription of imprinted genes. Although nucleolar positioning has been proposed as a mechanism to related to gene silencing, we find that silencing and perinucleolar localization through the *Kcnq1ot1* noncoding RNA are separable events.

Studies of the imprinted genes of mammals have demonstrated the underlying complexity of gene regulation and have helped to identify the numerous epigenetic mechanisms that we now have broad and significant roles both in normal development and in human disease. In addition to mechanisms of covalent modifications to the DNA and associated histones, structural regulation of the chromatin fiber itself is known to be important for proper imprinted expression of numerous loci. The formation of chromatin loops and hubs, compartmentalization of coregulated loci, and changes to chromatin condensation are known to participate in the process by which imprinting signals give rise to parent-of-origin differences in gene expression between alleles (Reik *et al.* 2004; Li *et al.* 2008; Terranova *et al.* 2008; Redrup *et al.* 2009; Sandhu *et al.* 2009). Recent work has implicated a role for the nucleolar periphery in the epigenetic state of the inactive X chromosome and the paternal allele of the *Kncq1* imprinted domain (Zhang *et al.* 2007; Mohammad *et al.* 2008; Pandey *et al.* 2008). However, because numerous epigenetic mechanisms participate in both events, it is unclear which involve perinucleolar localization.

On the paternal allele of the ∼1-Mb *Kcnq1*-imprinted domain, the *Kcnq1ot1* noncoding RNA (ncRNA) is required for repression of all neighboring imprinted genes (Fitzpatrick *et al.* 2002; Kanduri *et al.* 2002; Thakur *et al.* 2003, 2004; Mancini-Dinardo *et al.* 2006); see Supporting Information, Figure S1). Silencing of placentally imprinted genes involves the function of several histone methyltransferase systems, including G9a and both polycomb complexes (Pandey *et al.* 2008; Terranova *et al.* 2008; Wagschal *et al.* 2008; Redrup *et al.* 2009). As a regulatory ncRNA, *Kcnq1ot1* is thought to direct the action of these enzymes to the promoters of placentally imprinted genes and trigger compaction of this locus into a conformation refractory to pol II transcription (Kanduri 2011). This domain is also more frequently associated with the nucleolus in cells of a 14.5-dpc placenta compared with nuclei from cells of an embryonic derivative. It has been hypothesized that this localization reflects a mechanism for maintaining a heterochromatic state unique to extraembryonic tissue (Pandey *et al.* 2008).

Despite these findings, a number of questions remain. Among them is whether localization is a late event in the silencing of placental genes in this domain, similar to what has been observed for the inactive X chromosome in differentiating embryonic stem cells or, alternatively, whether localization precedes silencing, positioning the locus in a transcriptionally repressive nuclear compartment, where heterochromatic marks will be established and maintained at gene units encapsulated by an accumulation of *Kcnq1ot1* ncRNA. As the list of genes with tissue-specific imprinting patterns grows—especially those linked to human disease—as does the need to understand the mechanistic basis for this form of regulation.

We therefore sought to develop and *ex vivo* system for studying the establishment and maintenance of tissue specific, parent-of-origin expression patterns. In particular, those that use mechanisms affecting the structure and organization of chromatin fibers. In agreement with published observations *in vivo*, we have observed frequent localization of the paternal *Kcnq1* domain to the nucleolar periphery in mouse trophoblast stem (TS) cells. Differentiation induces a significant increase in association of *Kcnq1ot1* RNA domains with the nucleolus, diminished in the absence of full-length *Kcnqot1*. Surprisingly, *Kcnq1ot1* RNA foci are associated with the nucleolus in TS cells lacking a stable functional PRC2 complex, where multiple placentally imprinted genes within this locus are highly expressed. Moreover, active paternal alleles in EED-deficient TS cells can be readily observed by RNA fluorescence *in situ* hybridization (FISH) at the nucleolar periphery. Together, these results suggest *Kcnq1ot1*-dependent localization to the nucleolar periphery in TS cells may not be related to silencing of the placentally imprinted genes in this domain, and more broadly, localization to this nuclear compartment is not sufficient to preclude transcription.

## Materials and Methods

### Mouse strains and animal work

Mice carrying a deletion of the *Kcnq1ot1* promoter (*Kcnq1ot1*^termΔ^) were obtained from John Levorse and Shirley Tilghman of Princeton University (Mancini-Dinardo *et al.* 2006). Mice were genotyped using primers TermF (GTGCCCTAGGACACCGGCTCAGGCC) and TermR (CCTTCACAAAGATCCCTCGAGCCCAA).

### TS cell derivation and cell culture

TS cells of all genotypes were derived and maintained using standard conditions (Tanaka *et al.* 1998). Both WT and *Eed*^−/−^ TS cells were derived from F1 intercrosses of JF1 (maternal) and CD1 (paternal) animals. The *Kcnq1ot1*^+/termΔ^ TS lines were derived from crosses of *Kcnq1ot1*^termΔ/+^ males by C57BL6/J females. The *Eed*^−/−^ TS lines have been previously described (Kalantry *et al.* 2006). Differentiation was induced by removal of conditioned media and growth factors, and culture in RPMI medium with 20% ES-qualified FBS (Invitrogen) for 2 to 4 days.

### Immunofluorescence

TS cells were grown or differentiated on coverslips. Coverslips were permeabilized with cytoskeletal buffer (100 mM NaCl, 300 mM sucrose, 3 mM MgCl_2_, and 10 mM PIPES, pH 6.8), then fixed in 2% paraformaldehyde (PFA; Electron Microscopy Sciences) for 10 min at room temperature, washed twice for 5 min in 1× phosphate-buffered saline (PBS), and stored in 75% ethanol at 4°. Coverslips were rehydrated with several washes of 1× PBS, permeabilized with 0.25% Triton X-100 (Fisher) in 1× PBS, and then incubated for 30 to 60 min in Blocking Buffer: 5 mg/mL IgG-free bovine serum albumin (Jackson ImmunoResearch), 0.5% Tween-20 (Fisher), 1× PBS. Primary antibodies were diluted in Blocking Buffer and incubated overnight at 4°. The following primary antibodies (and dilutions) were used in this study: mouse-anti-CDX2 (AM392-5M; Biogenex), 1:50; mouse-anti-Fibrillarin (ab4566; Abcam), 1:50; rabbit-anti-Nucleolin (A300-711A; Bethyl); and rabbit anti-PCNA (sc-7907; Santa Cruz Biotechnologies), 1:200. After three 5-minute washes with 1× PBS, cells were incubated in secondary antibody diluted in Blocking Buffer, for 20 to 60 min at room temperature. The following secondary antibodies were used in this study, each at a 1:400 dilution: Alexafluor-568, goat-anti-rabbit (Invitrogen); Alexafluor-488, goat-anti-mouse (Invitrogen); biotynylated horse antimouse (Vector, BA-2001); and goat-anti-rabbit antibodies (Jackson ImmunoResearch, 111-065-144). DAPI (Invitrogen) was included in the secondary antibody incubation. After three 5-min washes in 1× PBS, cells were mounted in Vectashield (Vector) or SlowFade Gold(Invitrogen).

### FISH probes

The following probes were used in this study: BACs: *Kcnq1*, RP23-366M26 or RP24-271L24; fosmids: *Kcnq1ot1*, G135P604951G10, and G135P600002H11 (all clones were acquired from CHORI BPRC). The probe for *Cd81* was generated by amplifying the fragment directly from a cDNA pool (see Table S1 for primer information). BACs and fosmids were isolated by a standard alkaline lysis protocol. Approximately 25 ng of DNA was labeled with BioPrime DNA labeling kit (Invitrogen), using fluorescein isothiocyanate−conjugated dUTP (Roche) or Cy3-conjugated dCTP (GE Healthcare), and stored in 70% ethanol. To prepare FISH probes for hybridization, they were precipitated with mouse Cot-1 DNA (Invitrogen), yeast tRNA (Invitrogen), and Salmon Sperm DNA (Invitrogen). After washes with 75% and 100% ethanol, probes were air-dried and denatured for 10 min in 50 to 100 μL of 100% formamide. An equal volume of 2× hybridization buffer (25% dextran sulfate/4× SSC) was then added, and probes were prehybridized for 60 to 90 min at 37°. Probes were stored at −20° until use.

### FISH

Cells were dehydrated with washes of 85%, 95%, and 100% ethanol (3 min each), then air dried for 5 min. For DNA FISH, cells were denatured for 5 to 10 min at 85° in 70% formamide/2× SSC, then washed twice in cold 2× SSC. Prepared FISH probes were added to coverslips, and hybridized overnight at 37°. Coverslips were washed twice with prewarmed 50% formamide/2× SSC for 5 min each at 37°, followed by two washes with prewarmed 2× SSC for 5 min each at 37°. The RNA FISH protocol was identical except coverslips were not denatured. Finally, coverslips were mounted in Vectasheild with DAPI (Vector Labs), and visualized on a Leica DMLB flourescent microscope (Leica). Images were captured on a Retiga 2000R Fast camera (Qimaging), using QCapture software (Qimaging), and merged with Adobe Photoshop (Adobe). For RNA-DNA FISH experiments, RNA FISH was performed first, cells were refixed in 4% PFA, and then DNA FISH was performed as described. Only cells with two coplanar alleles and a clear nucleolus were scored in each field; a DNA FISH signal was considered to be perinucleolar if it fell within 5 pixels of the nucleolar edge (as determined by either DAPI or immunofluorescence against fibrillarin). Localization was analyzed in several independent WT and *Eed*^−/−^ TS lines, with similar results. For all FISH experiments, data from multiple hybridizations was pooled, and chi-squared analysis was used to determine significance.

### Combined immunofluorescence and FISH

Cells were fixed as described previously. For DNA FISH and DNA FISH combined with immunofluorescence for Fibrillarin, immunofluorescence was performed first as described previously but primary antibodies were detected with a 1:400 dilution of biotin-conjugated, donkey-antimouse or antirabbit secondary antibodies (Jackson ImmunoResearch). After three 5-min washes with 1× PBS, cells were refixed in 4% PFA for 10 min at room temperature. Coverslips were washed twice with 1× PBS, twice with 75% ethanol for 5 min each, then DNA was performed as described previously. To detect biotinylated secondary antibodies, coverslips were washed with once with 4× SSC for 5 min, incubated for 20 to 30 min in Streptavidin-647 or Streptavidin-568 (Invitrogen) in 2 mg/mL bovine serum albumin and 4× SSC, followed by 5 min washes of 4× SSC, 4× SSC with 0.5% Tween-20 (Fisher), and 4× SSC. All washes and incubations for biotin detection were carried out at 37°. Finally, cells were mounted with Vectashield with DAPI (Vector).

For RNA FISH, immunofluorescence for CDX2 was carried out first in RNAse-free conditions, detected with a biotinylated secondary antibody, then fixed for 3 min at room temperature with 2% PFA. Then, RNA FISH was carried out as described previously, and then fixed for 3 min at room temperature with 2% PFA. Finally immunofluorescence performed to detect Nucleolin and the biotinylated secondary antibody. Coverslips were mounted with SlowFade Gold (Invitrogen). Z-stacks of each channel were taken on a Ziess AxioImager M2 microscope, deconvolved using the Axiovision software package (Zeiss), then rendered in 3-dimensions and analyzed using the ZEN Light Edition 2009 software (Zeiss). Signals were considered associated with the nucleolus if they fell within 0.5 μm from their center to the nucleolar immunofluorescence signal. Statistical significance was determined with the χ^2^ test.

### cDNA synthesis and quantitative PCR

RNA was isolated from cultured cells with Trizol reagent (Invitrogen), and 1 μg of RNA was used for each reaction. Samples were reverse transcribed using Random-primer mix (NEB), and MultiScribe reverse transcriptase (Invitrogen). Real-time PCR was carried out with 20 to 40 ng of cDNA per PCR reaction, on a CFX96 real-time system (Bio-Rad) using SsoFast EvaGreen Supermix (Bio-Rad). Reactions were repeated in triplicate and normalized against expression of *Rpl19*. To determine PCR efficiency and specificity for allelic assays, a dilution series was generated using gel-isolated (QIAGEN), strain-specific PCR products. By calculating and normalizing for PCR efficiency, we could compare JF1 and CD1 products for each gene. For *Cd81*, we identified a 5-bp, simple-sequence length polymorphism between JF1 and CD1. *Cd81* PCR products were resolved on a 20% polyacrylamide gel (National Diagnostics), stained with SYBR green (Invitrogen), imaged on a Biospectrum Imaging System (UVP), and quantified using ImageJ software (NIH). All primer sequences and conditions are in Table S1.

## Results and Discussion

**Figure 1:**
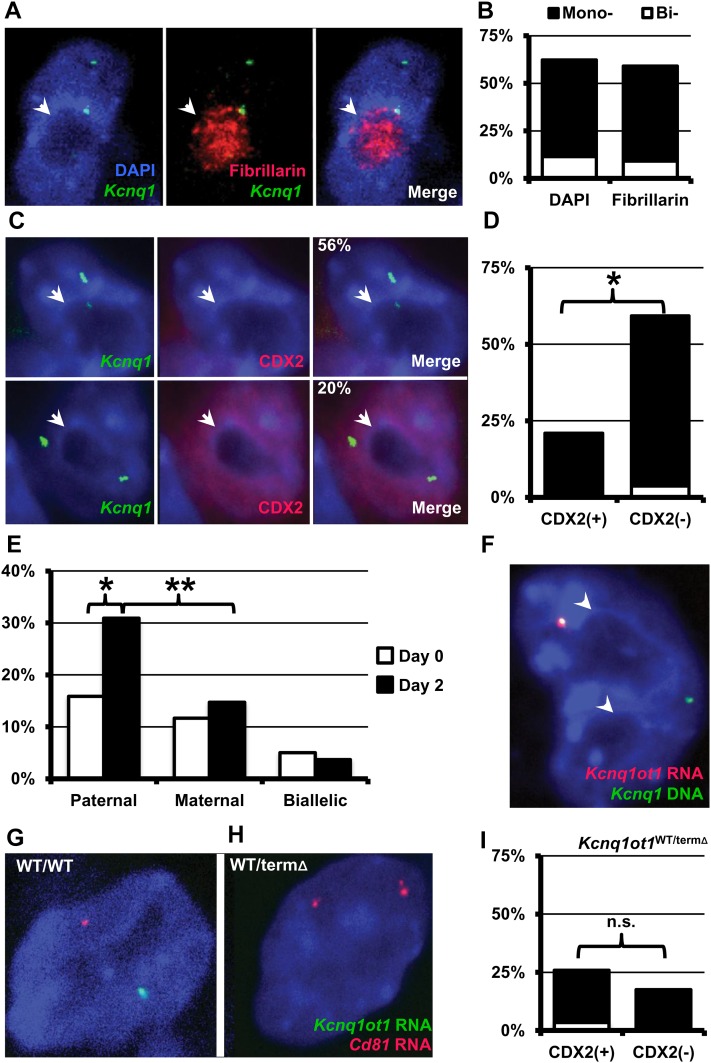
Localization of *Kcnq1* locus during TS differentiation. (A) Representative image of DNA FISH experiment (*Kcnq1* DNA,green) showing nucleolus counterstained with antibodies for the nucleolar protein, fibrillarin (red). (B) Measured frequency of localization of *Kcnq1* DNA FISH signals to the nucleolar periphery was not statistically different whether we used DAPI (*n* = 44) or fibrillarin (n = 61) to mark the nucleolus. (C) Representative images of Immunofluorescence/DNA FISH experiments, top row, are differentiated, CDX2-negative cells and bottom row are undifferentiated, CDX2-positive cells; the nucleolus is indicated by the white arrowhead. (D) Localization of the *Kcnq1* alleles to the nucleolar periphery in undifferentiated (CDX2+; n = 175) and differentiated (CDX2−; n = 67) WT TS cells. (E) Nucleolar assocaition of *Kcnq1ot1*-expressing alleles by RNA/DNA FISH in WT TS cells before (day 0, n = 120) and 2 days (day 2, n = 136) after the removal of growth factors to promote differentiation. (F) Representative image of RNA/DNA FISH experiment from E; *Kcnq1* DNA (green) and *Kcnq1ot1* RNA (red). RNA FISH IN WT (G) and *Kcnq1ot1*^WT/termΔ^ TS (H) cells, with probes for *Kcnq1ot1* (green) and *Cd81* (red). *Kcnq1ot1*^WT/termΔ^ TS cells had no detectable *Kcnq1ot1* expression, and biallelic expression of normally silent paternal alleles, including *Cd81*. (I) Nucleolar association of the *Kcnq1* alleles (DNA FISH) in CDX2+ (n = 91) and CDX2− (n = 40) *Kcnq1ot1*^WT/termΔ^TS cells. In all images, the nucleoli are indicated with white arrowheads. Unless otherwise indicated, white bars are cells with biallelic associations (Bi-) with the nucleoli; black bars show the proportion of cells with a mono-allelic association (Mono-). X-axis for all graphs are proportion of cells with a FISH signal found at the nucleolar periphery; (*), *P* < 10^−4^; (**), *P* < 10^−6^; n.s., not significant. Statistical significance was determined by the χ^2^ test.

We first analyzed the positions of the endogenous *Kcnq1* alleles by DNA FISH in wild-type (WT) TS cells by using DAPI staining patterns to identify the nucleolar periphery. TS cells have relatively flat nuclei with one to two large nucleoli, making them amenable for this type of analysis. In control experiments, no significant difference in nucleolar association of *Kcnq1* DNA was observed between experiments whether we identified the nucleolus with immunofluorescence against fibrillarin or lack of DAPI staining (Figure 1, A and B). We combined FISH experiments with immunofluorescence against the caudal-like transcription factor CDX2, expressed exclusively in undifferentiated TS cells (Ralston and Rossant 2005). A single *Kncq1* DNA signal was perinucleolar in approximately 20% of undifferentiated (CDX2-positive) TS cells, but 56% of CDX2-negative cells had a single perinucleolar *Kcnq1* allele (Figure 1, C and D, *P* < 0.0001). RNA-DNA FISH for *Kcnq1ot1* RNA and *Kcnq1* DNA in undifferentiated TS cells, and those cultured without growth factors and conditioned media for two days to promote differentiation, revealed the *Kcnq1ot1* ncRNA-associated allele was found significantly more frequently at the periphery of the nucleolus in the differentiated cells (Figure 1, E and F). The proportion of cells with a DNA only signal associated with the periphery (maternal), or cells with both RNA-DNA and DNA patterns at the nucleolar periphery (biallelic) did not significantly change during differentiation (Figure 1E). Thus, differentiation from the stem cell state appears to trigger an association of the paternal *Kcnq1* locus with the nucleolus.

*Kcnq1ot1*-expressing episomes have been shown to localize to the nucleolar periphery, coincident with silencing of reporter genes on the episomal construct (Mohammad *et al.* 2008). However, whether the localization of the endogenous locus is linked to this regulatory ncRNA is less clear, with current results potentially confounded by the closely linked, yet independently regulated *H19/Igf2* imprinted cluster. To definitively determine whether *Kcnq1ot1* is responsible for differentiation-induced nucleolar association of the endogenous locus, we derived TS cells carrying a paternal mutation of the *Kcnq1ot1* locus that terminates its transcription at 1.5 kb [*Kcnq1ot1*^WT/termΔ^ (Mancini-Dinardo *et al.* 2006)]. When inherited paternally, this mutation results in the expression of both ubiquitously and placentally imprinted genes from the normally silent allele. Because the imprinting of the *H19/Igf2* region is independent from that of *Kcnq1* domain (Caspary *et al.* 1998), any changes in localization observed in the *Kcnq1ot1*^WT/termΔ^ TS cells would be attributable to the lack of *Kcnq1ot1* expression or derepression of the surrounding genes on the mutant paternal chromosome. In agreement with a mechanistic link to the imprinting of the *Kcnq1* domain, the frequency of *Kcnq1ot1*^WT/termΔ^ TS cells with a nucleolus-associated *Kcnq1* DNA FISH signal did not increase upon differentiation (Figure 1, G−I). Thus, localization of this chromosomal domain is due to the expression of *Kcnq1ot1*, rather than the neighboring imprinted *H19/Igf2* region.

**Figure 2:**
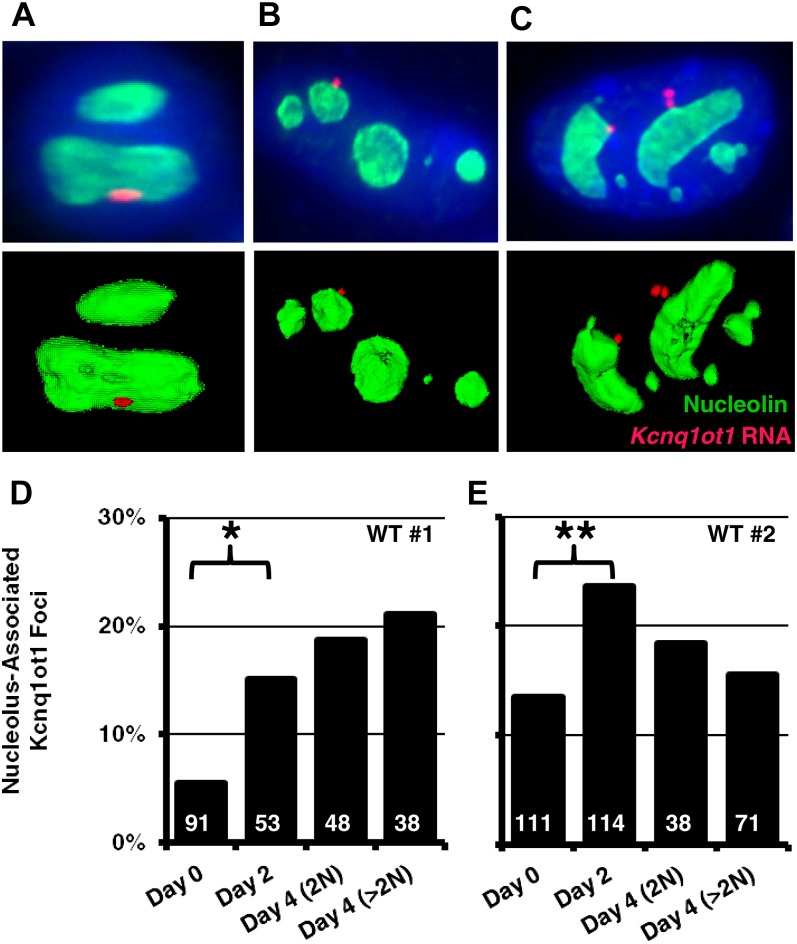
Dynamics of *Kcnq1ot1* ncRNA nucleolar association. Nucleolar association of *Kcnq1ot1* RNA FISH signal was measured to the edge of the nucleolus (as marked by nucleolin immunofluorescence) in three-dimensional reconstructions of single cells. Representative Z-stack projections (top) and three-dimensional reconstructions (bottom) illustrating examples of *Kcnq1ot1* nucleolar association in undifferentiated (A), differentiated (B), and endoreduplicated (C) cells. *Kcnq1ot1* RNA FISH is red, Nucleolin immunofluorescence is green. (D, E) Quantification of nucleolar associated RNA FISH foci in two independent WT lines. RNA FISH signals within 0.5 μm of edge of the Nucleolin signal were considered ‘nucleolus associated’. The number of RNA FISH foci analyzed (n) are shown within each bar; (*), *P* < 0.0007; (**), *P* < 0.0028; n.s., not significant. Statistical significance was determined by the χ^2^ test.

To accurately quantify the dynamic changes in localization of the paternal *Kcnq1* domain, we analyzed its position relative to the nucleolus in undifferentiated cells, along with several timepoints of differentiation. For this series of experiments, we used RNA FISH for the *Kcnq1ot1* ncRNA to mark the paternal allele, and immunofluorescence for the nucleolar protein, Nucleolin, to mark the nucleolus. Deconvolved Z-stack images were used to generate three-dimensional reconstructions of nucleoli and FISH signals, which were used to quantify distance. We used a stringent cutoff for nucleolar association (within 0.5 μm, from the center of the FISH signal to the edge of the Nucleolin signal, Figure 2, A−C). To accurately compare undifferentiated cells, we used immunofluorescence for CDX2. In the undifferentiated state, localization frequency varied between two independently derived, WT TS lines (Figure 2, D and E). However, in both cases, nucleolar association of *Kcnq1ot1*-expressing alleles increased approximately twofold in after the removal of conditioned media and growth factors. Thus, nucleolar association of the paternal *Kcnq1* allele increases as TS cells differentiate.

Although the mature placenta is made of several specialized cell types, some imprinted genes only show significant allelic expression biases in a subpopulation of these tissues (Hudson *et al.* 2010). In culture, removal of conditioned media and growth factors from TS cells results in the preferential formation of trophoblast giant cells [TGCs (Hughes *et al.* 2004)]. TGCs, which serve essential roles to support placental development *in vivo*, undergo numerous rounds of endoreduplication as they differentiate, forming large, polyploid nuclei. Unique in this regard, we hypothesized that as TGCs continue to undergo successive rounds of endoreduplication, paternal *Kcnq1* loci may accumulate at the nucleolar periphery. We compared the localization of *Kcnq1ot1* foci in diploid cells (cells with 1 *Kcnq1ot1* RNA FISH signal) to polyploid cells (>1 *Kcnq1ot1* focus). After 4 days of differentiation, most cells in both WT cultures were visibly polyploid, with an average genome content of 12N (data not shown). Surprisingly, the frequency of a locus to be associated with the nucleolus was no different in polyploid cells compared with differentiated diploid cells at days 2 or 4 (Figure 2, D and E). Although nucleolar association of the *Kcnq1ot1* ncRNA appears to be more common in differentiating TS cells, these observations suggest that this position does not reflect a permanent compartmentalization in TGCs.

**Figure 3:**
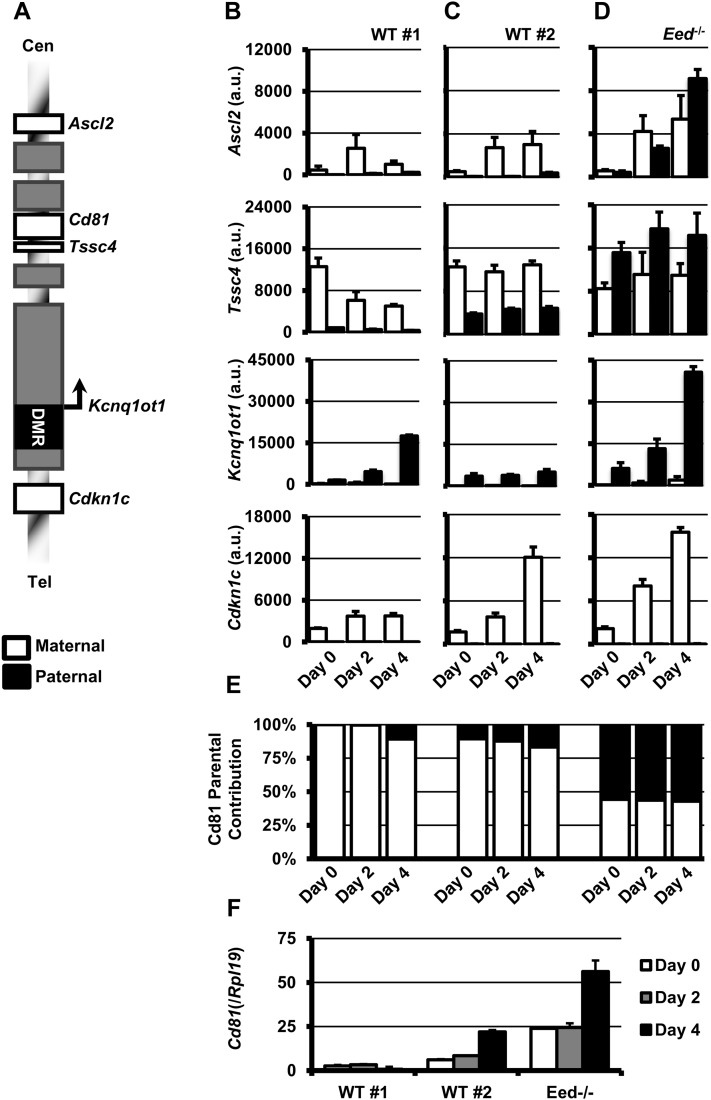
Allelic expression of imprinted genes in the *Kcnq1* domain during TS cell differentiation. Allelic expression of 3 placentally imprinted genes (*Ascl2*, *Cd81*, and *Tssc4*), one ubiquitously imprinted gene (*Cdkn1c*), as well as the paternally expressed *Kcnq1ot1* ncRNA were analyzed in undifferentiated and differentiated TS lines. For *Ascl2*, *Tssc4*, *Kcnq1ot1*, and *Cdkn1c*, PCR efficiency was first determined for each allelic PCR assay to be able to then compare CD1 and JF1 products (See *Materials and Methods* and Figure S2 for additional information). (A) Schematic of the Kcnq1 locus showing relative positions of genes analyzed in this study. Gray boxes represent genes whose expressed was not detectable in undifferentiated or differentiated time points or expressed but lacking SNPs to distinguish parental alleles. Expression of maternal (JF1, white bars) and paternal (CD1, black bars) alleles in two WT TS lines (B,C) and an Eed-deficient TS line (*Eed*^−/−^; D) in undifferentiated (day 0), and after 2 and 4 days of differentiation. Expression levels of Cd81 (E) and relative amount of maternal and paternal product in each sample (F). Parental contribution for (F) was determined on a nondenaturing 20% PAGE gel to resolve SSLP between JF1 and CD1 products.

In addition to imprinted expression limited to specific tissue subpopulations, differences in allelic expression levels of imprinted genes can be manifest during the differentiation from the stem cell state (Latos *et al.* 2009). Therefore, we hypothesized that the change in localization of the paternal *Kcnq1* allele may be related to changes in allelic biases of expression of placentally imprinted genes. To determine how differentiation and a change in localization affect their transcription, we analyzed allele-specific expression of the placentally imprinted genes during the course of TS cell differentiation. As the WT lines were F1 hybrids from JF1 (maternal) and CD1 (paternal) crosses, we only analyzed genes were we could identify SNPs to discern the parent-of-origin (Figure 3A). In addition, we excluded *Th* and *Tspan32*, as their transcript levels were low/below the limit of detection (data not shown). We included analysis of the *Kcnq1ot1* ncRNA and the ubiquitously imprinted gene, *Cdkn1c*. For each gene (except *Cd81*), we designed primers around SNPs to detect specifically JF1 or CD1 transcript, optimizing conditions to give at least 10-fold enrichment against the nonspecific allele (Figure S2). In most cases, specificity of the qPCR assay for the correct allele was determined to be >20-fold. For *Cd81*, we identified a short-sequence length polymorphism that we used to discern the JF1 and CD1 alleles after quantitative PCR on a nondenaturing polyacrylamide gel (see Figure S3).

As expected, *Ascl2*, *Tssc4*, and *Cd81* were all predominantly expressed from the maternal allele in both WT lines we analyzed (Figure 3, B and C, E and F). Although one control line had significantly more paternal contribution, the ratio of maternal to paternal product did not change with differentiation progressed, even as overall levels appeared to be dynamically regulated. However, these ratios remained constant even as localization frequency increased. Thus, parent-of-origin expression patterns are already established in the undifferentiated state of TS cells, and do not appear affected by differentiation *in vitro*. Importantly, increased nucleolar association in differentiated cell types did not result in observable differences in levels of the paternal alleles of imprinted genes in this domain.

Tissue-specific imprinting of several genes within the *Kcnq1* domain requires the activities of several histone methyltransferases, including polycomb repressive complex 2 [PRC2 (Pandey *et al.* 2008; Terranova *et al.* 2008)]. To determine whether nucleolar association was related to the repression of the placentally imprinted genes by PRC2, we analyzed localization of the *Kcnq1* alleles in TS cells carrying a loss-of-function mutation of the PRC2 component, EED. Loss of EED results in destabilization of all other PRC2 components, including the catalytic component EZH2, and significant reduction of global H3K27me3 (Montgomery *et al.* 2005). Importantly, this results in the paternal expression of placentally imprinted genes within the *Kcnq1* domain (Mager *et al.* 2003; Lewis *et al.* 2006). In contrast to the control cell lines, robust paternal expression was observed for *Ascl2*, *Tssc4*, and *Cd81*, in both undifferentiated and differentiated timepoints (Figure 3, D−F). However, differentiation did not affect the ratio of maternal to paternal product for each of these placentally imprinted genes.

**Figure 4:**
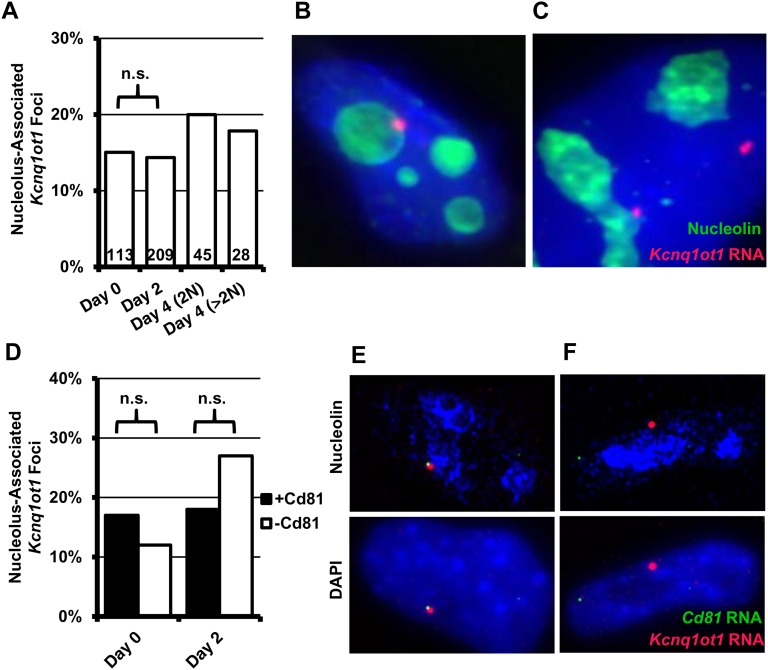
Localization of *Kcnq1ot1* RNA foci and active paternal *Cd81* in *Eed*^−/−^ TS cells. (A) Summary of *Kcnq1ot1* RNA localization in undifferentiated and differentiated *Eed*^−/−^ TS cells. Representative images (Z-stack projections) of undifferentiated TS (B) and endoreduplicated (C) Eed-null cells (DAPI, blue; Nucleolin, green; *Kcnq1ot1* ncRNA, red). (D) To determine whether paternal expression of placentally imprinted genes was compatible with nucleolar association, we compared localization frequency of *Kcnq1ot1* RNA foci overlapping *Cd81* RNA FISH signals (black bars) against *Kcnq1ot1* foci without detectable *Cd81* (open bars) in undifferentiated and differentiated *Eed*^−/−^ TS cells. Nucleolar association of paternal *Cd81* RNA FISH signals in *Eed*^−/−^ TS was not statistically different from localization of *Kcnq1ot1* RNA foci. Representative images of cells with overlapping *Kcnq1ot1* (red) and *Cd81* (green) RNA FISH signals, which we counted as +*Cd81*, (E) and without, which were counted as −*Cd81* (F). Top shows RNA FISH with nucleolin immunofluorescence; bottom with DAPI staining. For panels (A) and (D), the total number of RNA FISH foci analyzed (n) are shown within each bar. Statistical significance was determined by χ-squared tests; n.s., not significant.

Because imprinted expression was affected in *Eed*^−/−^ TS cells, we speculated that nucleolar association of the *Kcnq1* domain may be reduced or entirely absent in these cells. In undifferentiated EED-deficient TS cells, localization of *Kcnq1ot1* to the nucleolar periphery was similar to that in undifferentiated, WT TS cells (Figure 4, A and B). However, in contrast to the increase observed in both WT lines, the frequency of localization did not change significantly during the time course of differentiation, or in the few endoreduplicated cells that appeared as differentiation progressed (Figure 4, A and C). However, the number of *Eed*^−/−^ TS cells undergoing DNA synthesis (as measured by staining for proliferating cell nuclear antigen) after the induction of differentiation was significantly reduced compared with the WT (10%, *Eed*^−/−^ *vs.* 50%, WT, Figure S4). Thus, this may be an indirect result of little-to-no proliferation in the differentiating *Eed*^−/−^ TS line. Nevertheless, localization of the *Kcnq1ot1* RNA domain is not completely ablated in the absence of PRC2. Furthermore, these results suggest that the association of the *Kcnq1ot1*-expressing allele with the nucleolus may not be related to the silencing of the placentally imprinted genes at this locus.

To determine whether expression is compatible with nucleolar association, we analyzed expression of the active paternal alleles in *Eed*^−/−^ TS cells and differentiated derivatives by RNA FISH, together with *Kcnq1ot1* RNA FISH. *Cd81* is not only biallelic in *Eed*^−/−^ TS cells, but its robust up-regulation during differentiation made it technically compatible with this experiment. In contrast, we could not detect *Ascl2* or *Tssc4* by RNA FISH (data not shown). To detect *Cd81* transcript, we used a portion of the cDNA that did not overlap the antisense *R74862* transcript. We expected that if expression of paternal *Cd81* was not compatible with a position near the nucleolar periphery, then we should only observe overlapping *Cd81*/*Kcnq1ot1* RNA FISH signals away from this region. However, the frequency of *Cd81*/*Kcnq1ot1* RNA FISH signals associated with the nucleolus was not statistically different from the nucleolar association of *Kcnq1ot1* foci without *Cd81*. This observation suggests compartmentalization of this locus near the nucleolus is not sufficient to preclude transcription of linked protein coding genes.

Changes to the organization of chromatin within the nucleus accompany many developmental transitions and have been observed in cancers and hereditary human diseases. These changes are thought to represent functional events, most often related to the transcriptional activity of genes. However, the mechanistic basis for these changes is poorly understood. Previous work has suggested that the paternal *Kcnq1* allele is associated with the nucleolus in extraembryonic tissue, and postulated that this localization pattern was related to maintaining a heterochromatic state specifically at the placentally imprinted genes (Pandey *et al.* 2008). Here, we have used a cell-culture system to explore the mechanistic requirements for this phenomenon. We show that nucleolar association significantly increases in frequency upon differentiation of TS cells, and is dependent upon expression of full-length *Kcnq1ot1*. Interestingly, this locus does not become fixed at the nucleolar periphery in endoreduplicating trophoblast giant cells.

In EED-deficient TS cells, were the paternal alleles of placentally imprinted genes in the *Kcnq1* domain are highly expressed, *Kcnq1ot1*-expressing loci did associate with the nucleolus; however, this frequency did not increase during differentiation. This latter observation may be an indirect effect: *Eed*^−/−^ embryos are known to have defects in the development of extraembryonic tissues(Wang *et al.* 2002), and we observed that proliferation in *Eed*^−/−^ TS cells is significantly reduced (Figure S4). Importantly, RNA FISH signals for *Cd81*, overlapping those of *Kcnq1ot1*, were frequently found at the nucleolar periphery. Although we cannot rule out a repressive effect at all imprinted genes, this observation suggests that association with the nucleolar periphery is not itself sufficient to silence the placentally imprinted subset of genes within this domain.

The increased frequency of this phenomenon concurrent with the onset of differentiation suggests a functional role related to the imprinting by, or expression of, the *Kcnq1ot1* ncRNA. This role may be limited to specific extraembryonic cell populations, where association with the nucleolus may augment, or perhaps even attenuate, Kcnq1ot1 function on a subset of elements or genes. This may involve genes which we could not analyze due to the lack of an informative SNP, or perhaps a nearby gene not currently known to be imprinted. Indeed, conflicting reports have emerged as to the imprinted status of a number of genes in this cluster (reviewed in Proudhon and Bourc’his 2010; Lefebvre 2012), some of which may only be imprinted at specific developmental timepoints and within defined cell types. Although TS cells can differentiate into a number of extraembryonic lineages, TGCs appear to be the default pathway *in vitro* (Cross 2005). However, TGCs themselves are not a homogenous cell type, as discrete populations of polyploid cells within the placenta exist *in vivo* to carry out specialized functions(Hu and Cross 2010). Thus, nucleolar-associated *Kcnq1ot1* may not be widespread event in all placental tissues, but rather a hallmark of a select cell type.

## Supplementary Material

Supporting Information
