# Supplementary material for: Differentiation-Driven Nucleolar Association of the Mouse Imprinted Kcnq1 Locus
Source: G3 (Bethesda). 2012 Dec 1;2(12):1521–8. doi: 10.1534/g3.112.004226 (PMC3516474; doi:10.1534/g3.112.004226)
Supplement: Supporting Information [file supp_2_12_1521__index.html]

Supporting Information 

# Differentiation-Driven Nucleolar Association of the Mouse Imprinted *Kcnq1* Locus

## Supporting Information for Fedoriw *et al.*, 2012

**Files in this Data Supplement:**

- Supporting Information - Figures S1-S4 and Table S1 (PDF, 217 KB)
- Figure S1 - Schematic of the imprinted *Kcnq1* cluster (PDF, 76 KB)
- Figure S2 - Specificity of allele-specific qPCR assays (PDF, 103 KB)
- Figure S3 - *Cd81* allelic assay (PDF, 99 KB)
- Figure S4 - Proliferation of WT and *Eed*-deficient cells (PDF, 83 KB)
- Table S1 - RT-PCR primers and allele specific assays (PDF, 64 KB)
