# Supplementary material for: Differentiation-Driven Nucleolar Association of the Mouse Imprinted Kcnq1 Locus
Source: G3 (Bethesda). 2012 Dec 1;2(12):1521–8. doi: 10.1534/g3.112.004226 (PMC3516474; doi:10.1534/g3.112.004226)
Supplement: Supporting Information [file supp_2.12.1521_004226SI.pdf]

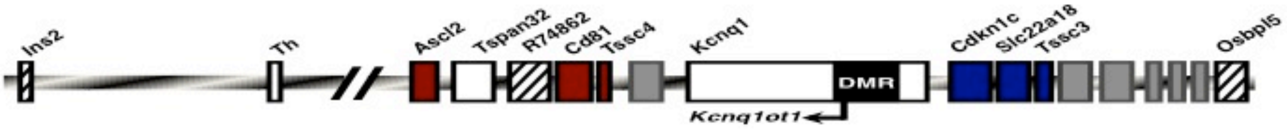

Figure S1 Schematic of the imprinted *Kcnq1* cluster. A paternally hypomethylated differentially methylated region (KvDMR1, black box) serves as an active promoter for the *Kcnq1ot1* ncRNA. The ubiquitously imprinted genes are in blue (?); genes which have not been reported as imprinted are in grey. Placentally-imprinted genes analyzed in this study are in red. *Kcnq1, Th,* and *Tspan32* were not expressed in TS cells or their differentiated derivatives (open boxes). *Ins2*, *R74862*, and *Osbpl5* were found expressed in TS cells; however, we found no SNP to discriminate between JF1 and CD1 alleles (striped boxes).

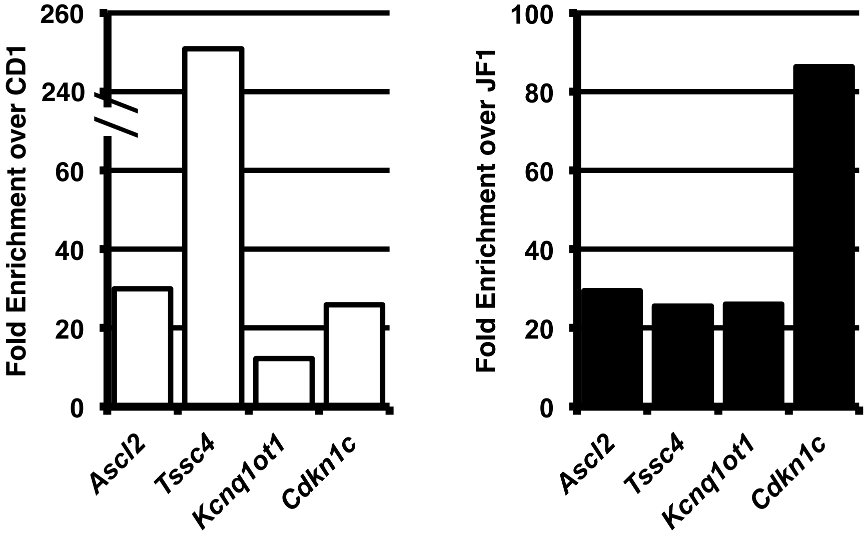

Figure S2 Specificity of allele-specific qPCR assays. Fold enrichment of JF1 (A), and CD1 (B) qPCR assays. For each experiment, 10^5 copies of CD1 and JF1 PCR product were used as template. Bar represents average of 2-4 replicates.

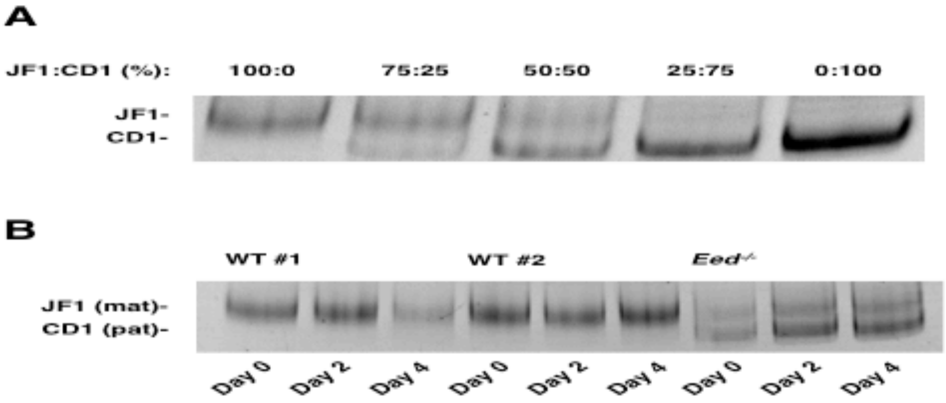

Figure S3 *Cd81* allelic assay. (A) Non-denaturing PAGE of *Cd81* amplicons, using various ratios of CD1 and JF1 gel-purified template as starting material (10^5 copies/PCR). The CD1 product consistently gave stronger bands. (B) Representative 20% PAGE of *Cd81* qPCR experiment (from Figure 3).

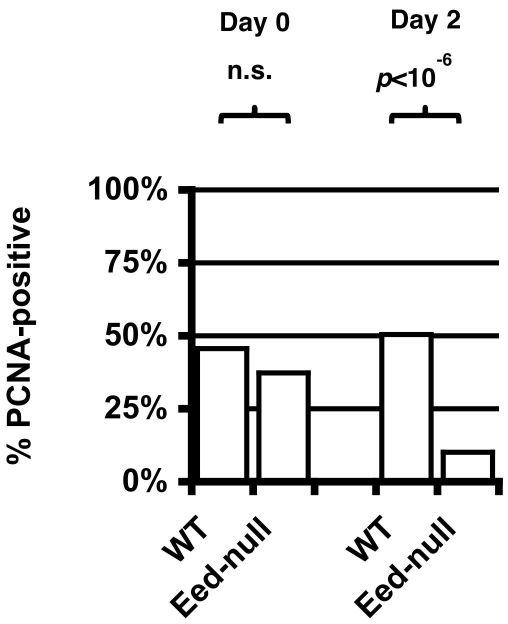

Figure S4 Proliferation of WT and *Eed*-deficient cells. Immunoflouresence was performed on coverslips of designated genotypes and timepoints. Statistical significance was determined by chi-squared. The (n) for each experiment: WT, day 0 (n = 112); WT, day 2 (n = 141); *Eed*$^{-/-}$, day 0 (n = 110); *Eed*$^{-/-}$, day 2 (n = 79).

Table S1 RT-PCR primers and allele specific assays. SNPs are boldfaced and underlined.

Gene	Strain	Fwd/Rev	Sequence
Ascl2	JF1	Fwd	TTCAGTAGAGTCCTACA<u>**G**</u>C
	CD1	Fwd	TTCAGTAGAGTCCTACA<u>**A**</u>C
		Rev	ATCTTCCATCTTCCGGACCT
Tssc4	JF1	Fwd	ACAGACAGCCCACACCTTC<u>**T**</u>
	CD1	Fwd	ACAGACAGCCCACACCTTC<u>**C**</u>
		Rev	CTCTGCTCCCCAAACCAGT
Cd81	both	Fwd	GGGGACATGGCCTGTGTAT
		Rev	CCCATGTGTGATGTCAGCTC
Kcnq1ot1	JF1	Fwd	CGATCTGCCTCAGCAAT<u>**C**</u>
	CD1	Fwd	CGATCTGCCTCAGCAAT<u>**T**</u>
		Rev	CTGAGAAGCCAAGTGGATCG
Cdkn1c	JF1	Fwd	AGATCTGACCTCAGACCCA<u>**G**</u>
	CD1	Fwd	AGATCTGACCTCAGACCCA<u>**A**</u>
		Rev	ACCTGCTCAGGGACCTGTT
Rpl19	N/A	Fwd	GGCCCAAGCCGATTTCAGA
		Rev	TCAGGAACCTTCTCTCGTCTTC
Cd81 (cDNA RNA FISH probe)		5′ Fwd	TGCAGTAAGGGGGTGAGTATG
		5′ Rev	GCGTCCTTGCTTCAAAGAGA
		3′ Fwd	TTCCATGAGACGCTCAACTG
		3′ Rev	CCCATTAGCATGCCTGATTT
