# Supplementary material for: Differentiation-Driven Nucleolar Association of the Mouse Imprinted Kcnq1 Locus
Source: G3 (Bethesda). 2012 Dec 1;2(12):1521–8. doi: 10.1534/g3.112.004226 (PMC3516474; doi:10.1534/g3.112.004226)
Supplement: Supporting Information [file supp_2.12.1521_FigureS1.pdf]

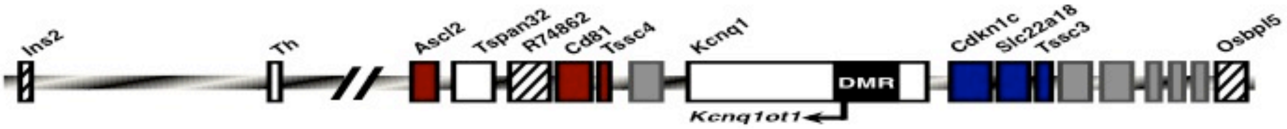

Figure S1 Schematic of the imprinted *Kcnq1* cluster. A paternally hypomethylated differentially methylated region (KvDMR1, black box) serves as an active promoter for the *Kcnq1ot1* ncRNA. The ubiquitously imprinted genes are in blue (?); genes which have not been reported as imprinted are in grey. Placentally-imprinted genes analyzed in this study are in red. *Kcnq1, Th,* and *Tspan32* were not expressed in TS cells or their differentiated derivatives (open boxes). *Ins2*, *R74862*, and *Osbpl5* were found expressed in TS cells; however, we found no SNP to discriminate between JF1 and CD1 alleles (striped boxes).
