# Supplementary material for: Differentiation-Driven Nucleolar Association of the Mouse Imprinted Kcnq1 Locus
Source: G3 (Bethesda). 2012 Dec 1;2(12):1521–8. doi: 10.1534/g3.112.004226 (PMC3516474; doi:10.1534/g3.112.004226)
Supplement: Supporting Information [file supp_2.12.1521_FigureS2.pdf]

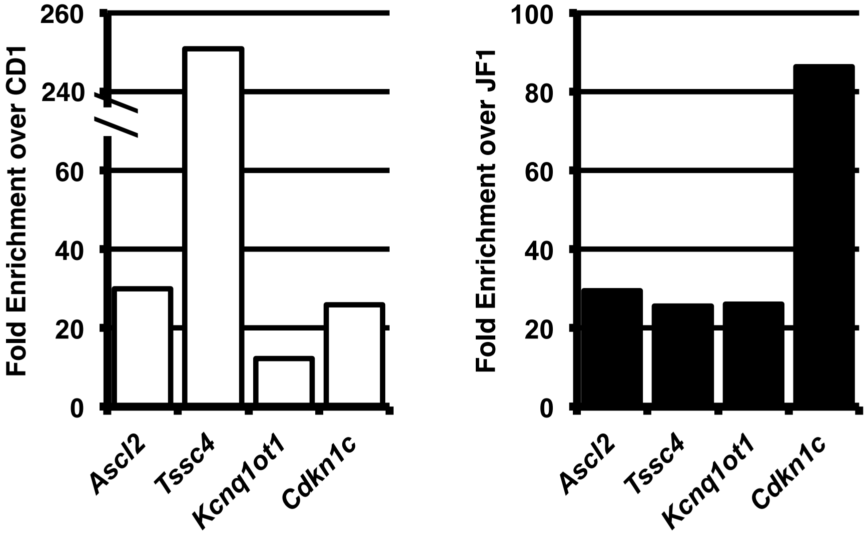

Figure S2 Specificity of allele-specific qPCR assays. Fold enrichment of JF1 (A), and CD1 (B) qPCR assays. For each experiment, 10^5 copies of CD1 and JF1 PCR product were used as template. Bar represents average of 2-4 replicates.
