# Supplementary material for: Differentiation-Driven Nucleolar Association of the Mouse Imprinted Kcnq1 Locus
Source: G3 (Bethesda). 2012 Dec 1;2(12):1521–8. doi: 10.1534/g3.112.004226 (PMC3516474; doi:10.1534/g3.112.004226)
Supplement: Supporting Information [file supp_2.12.1521_FigureS3.pdf]

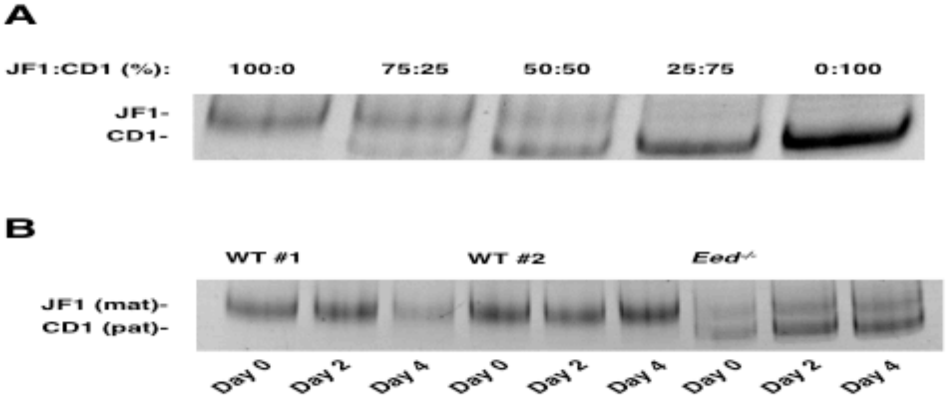

Figure S3 *Cd81* allelic assay. (A) Non-denaturing PAGE of *Cd81* amplicons, using various ratios of CD1 and JF1 gel-purified template as starting material (10^5 copies/PCR). The CD1 product consistently gave stronger bands. (B) Representative 20% PAGE of *Cd81* qPCR experiment (from Figure 3).
