# Supplementary material for: Differentiation-Driven Nucleolar Association of the Mouse Imprinted Kcnq1 Locus
Source: G3 (Bethesda). 2012 Dec 1;2(12):1521–8. doi: 10.1534/g3.112.004226 (PMC3516474; doi:10.1534/g3.112.004226)
Supplement: Supporting Information [file supp_2.12.1521_FigureS4.pdf]

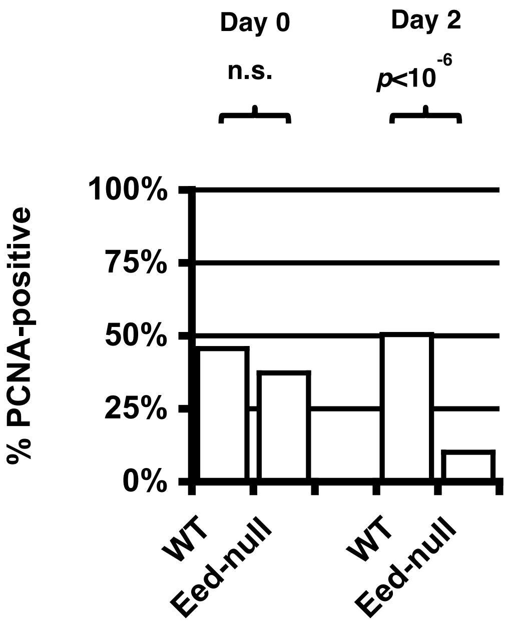

Figure S4 Proliferation of WT and *Eed*-deficient cells. Immunoflouresence was performed on coverslips of designated genotypes and timepoints. Statistical significance was determined by chi-squared. The (n) for each experiment: WT, day 0 (n = 112); WT, day 2 (n = 141); $Eed^{-/-}$, day 0 (n = 110); $Eed^{-/-}$, day 2 (n = 79).
