# Supplementary material for: Differentiation-Driven Nucleolar Association of the Mouse Imprinted Kcnq1 Locus
Source: G3 (Bethesda). 2012 Dec 1;2(12):1521–8. doi: 10.1534/g3.112.004226 (PMC3516474; doi:10.1534/g3.112.004226)
Supplement: Supporting Information [file supp_2.12.1521_TableS1.pdf]

Table S1 RT-PCR primers and allele specific assays. SNPs are boldfaced and underlined.

Gene	Strain	Fwd/Rev	Sequence
Ascl2	JF1	Fwd	TTCAGTAGAGTCCTACA**G**C
	CD1	Fwd	TTCAGTAGAGTCCTACA**A**C
		Rev	ATCTTCCATCTTCCGGACCT
Tssc4	JF1	Fwd	ACAGACAGCCCACACCTTC**T**
	CD1	Fwd	ACAGACAGCCCACACCTTC**C**
		Rev	CTCTGCTCCCCAAACCAGT
Cd81	both	Fwd	GGGGACATGGCCTGTGTAT
		Rev	CCCATGTGTGATGTCAGCTC
Kcnq1ot1	JF1	Fwd	CGATCTGCCTCAGCAAT**C**
	CD1	Fwd	CGATCTGCCTCAGCAAT**T**
		Rev	CTGAGAAGCCAAGTGGATCG
Cdkn1c	JF1	Fwd	AGATCTGACCTCAGACCCA**G**
	CD1	Fwd	AGATCTGACCTCAGACCCA**A**
		Rev	ACCTGCTCAGGGACCTGTT
Rpl19	N/A	Fwd	GGCCCAAGCCGATTTCAGA
		Rev	TCAGGAACCTTCTCTCGTCTTC
Cd81 (cDNA RNA FISH probe)		5’ Fwd	TGCAGTAAGGGGGTGAGTATG
		5’ Rev	GCGTCCTTGCTTCAAAGAGA
		3’ Fwd	TTCCATGAGACGCTCAACTG
		3’ Rev	CCCATTAGCATGCCTGATTT
